# Programmed death-ligand 1 expression associated with molecular characteristics in surgically resected lung adenocarcinoma

**DOI:** 10.1186/s12967-016-0943-4

**Published:** 2016-06-24

**Authors:** Zhengbo Song, Xinmin Yu, Guoping Cheng, Yiping Zhang

**Affiliations:** Department of Medical Oncology, Zhejiang Cancer Hospital, 38 Guangji Road, Hangzhou, 310022 People’s Republic of China; Key Laboratory Diagnosis and Treatment Technology on Thoracic Oncology, Hangzhou, 310022 Zhejiang People’s Republic of China; Department of Pathology, Zhejiang Cancer Hospital, Hangzhou, 310022 People’s Republic of China

**Keywords:** Non-small cell lung cancer, Programmed cell deathligand 1, Lung adenocarcinoma, Gene mutation, Coexisting mutations, Prognosis

## Abstract

**Background:**

Several clinical trials have shown that immune treatment focus on programmed death-1 and programmed death-ligand 1 (PD-L1) yields a good clinical efficacy in advanced non-small cell lung cancer (NSCLC). We investigated whether the PD-L1 expression was related to clinicopathologic and molecular characteristics in patients with surgically resected NSCLC.

**Methods:**

Between December 2008 and 2013, formalin-fixed, paraffin-embedded samples were obtained from patients with lung adenocarcinoma at Zhejiang Cancer Hospital. RT-PCR was used to analyze *EGFR, KRAS, NRAS, PIK3CA, BRAF, HER2* mutations and *ALK, ROS1, RET* fusion genes. The PD-L1 expression was evaluated by immunohistochemistry and staining of 5 % or more was scored as positive expression. Survival analysis was evaluated using the Kaplan–Meier method. Multivariate regression was performed using the Cox proportional hazards model.

**Results:**

Mutations were detected in 76.6 % of the 385 patients tested: *EGFR* mutation (n = 205, 53.2 %), followed by *EML4*–*ALK* rearrangement (n = 18, 4.7 %), *KRAS* (n = 16, 4.2 %), *HER2* (n = 9, 2.3 %), *ROS1* rearrangement (n = 8, 2.1 %), *PIK3CA* (n = 6, 1.6 %), *RET* rearrangement (n = 6,1.6 %), *BRAF* (n = 2, 0.5 %), and *NRAS* mutations (n = 1, 0.2 %). Twenty-four (6.2 %) patients carried coexisting mutations. PD-L1 expression was detected in 48.3 % (186/385) of all the patients. PD-L1 positive patients more frequently carried coexisting mutations (18/24, 75 %), followed by single-gene (145/271, 53.5 %) and pan-negative mutations (23/90, 25.6 %). PD-L1 expression decreased disease-free survival (DFS) in univariate analysis (*P* = 0.014). Multivariate analysis revealed that PD-L1 expression was not an independent risk factor for poor DFS and overall survival (OS) (*P* = 0.22 and 0.37, respectively).

**Conclusions:**

PD-L1 overexpression is more frequently observed in oncogene-mediated lung adenocarcinoma, especially with coexisting mutation subtypes. PD-L1 expression is not a prognostic factor in surgically resected lung adenocarcinoma patients.

## Background

Lung cancer is the leading cause of cancer-related death in China [1]. The standard treatment of lung cancer, especially non-small cell lung cancer (NSCLC) comprises platinum-based chemotherapy and driver gene-based targeted therapy, which resulted in extended survival and increased the quality of life in NSCLC patients [2–7]. However, drug resistance is a major challenge in most patients [8]. The median survival time in advanced NSCLC is no more than 2 years because of limited treatments available excluding chemotherapy and targeted therapy [9, 10].

Blockade of immune checkpoints in cancer with monoclonal antibodies has recently emerged as a promising approach to the treatment of solid tumors. Programmed death 1 (PD1), which belongs to the CD28 family of proteins, is a T cell surface receptor that regulates T cell activation and proliferation. Its ligand, programmed death-ligand 1 (PD-L1), is frequently expressed in many types of carcinomas [11–14]. Recent clinical trials found that inhibition of the PD-L1-PD1 interaction using specific antibodies resulted in promising antitumor efficacy in patients with various carcinomas [15, 16]. PD-L1 overexpression in NSCLC was reported ranging from 19 to 100 % [17–19]. Although several studies elucidated the association between common driver genes and PD-L1 expression in NSCLC, the results remain controversial and the prognostic value of PD-L1 expression is unclear [20].

This study focused on patients with completely resected lung adenocarcinoma and evaluated the association of PD-L1 expression with clinicopathologic parameters and driver genes, as well as its prognosis value in Chinese patients.

## Patients and methods

### Patients

A total of 385 adenocarcinoma patients underwent resection between December 2008 and 2013 in Zhejiang Cancer Hospital. Histological typing was determined according to the 2004 World Health Organization classification [21]. Tumor-node-metastasis (TNM) staging was based on the 7th edition of the lung cancer staging system. The recurrence or metastases were confirmed using chest CT, brain MRI, and bone scan as well as ultrasound and/or CT of the abdomen. The exclusion criteria included: (1) preoperative chemotherapy or radiation therapy, (2) death from other diseases unrelated to NSCLC. *The Ethics Committee of Zhejiang Cancer Hospital approved this study and written informed consent was obtained from each participant.*

### Immunohistochemical analysis of PD-L1 expression

Immunohistochemical (IHC) staining of PD-L1 expression was performed on 4-6 μm thick formalin-fiated, paraffi-embedded tissue. The concentration of rabbit primary antibody that reacts to PD-L1 The concentration of rabbit primary antibody that reacts to PD-L1 (**Proteintech Group Inc., Chicago, IL, USA**, Catalog number: 66248-1-Ig) was 1:100 in Dako antibody diluent; slides were incubated with this antibody overnight at 4 °C. Then, the slides were incubated with Ventana Omni Mapanti-rabbit secondary antibody for 60 min. AVentana Chromo MapKit was used for antibody detection, and then the slides were counterstained with hematoxylin. Next, the slides were dehydrated and cover slipped as per normal laboratory protocol. Two independent pathologists (Wei Wu and Guoping Cheng) assessed the expressions.

PD-L1 immunostaining results were classified into two groups based on the degree and intensity of staining: (1) negative, when staining was absent or detected in <5 % of the cells; and (2) positive, when membranous staining was present in ≥5 % of the cells.

We used another antibody (5H1, Cell Signaling Technology, Beverly, MA, USA) to confirmed the PD-L1 expression in 102 patients. The PD-L1 immunostaining criterion is same with the former antibody.

### Gene analysis

Genomic DNA or RNA was extracted from tumor tissues according to standard protocols (RNeasy Mini Kit, and QiAamp DNA Mini Kit, Qiagen, Hilden, Germany). Briefly, the isolated RNA samples were used for reverse transcription into cDNA using Revert Aid First Strand cDNA Synthesis Kit (Fermentas, St Leon-Rot, Germany). Either genomic DNA or cDNA was used for PCR amplification and sequencing. EGFR, HER2, KRAS, NRAS, BRAF, and PIK3CA were PCR amplified using genomic DNA. Cycle sequencing of the purified PCR products was carried out with PCR primers using the commercially available ADx Mutation Detection Kits (Amory, Xiamen, China).

The ALK, ROS1, and RET fusion mRNA was detected by PCR with fusion gene detection kit (Amory, Xiamen, China). In brief, total RNA was extracted with QiagenRNeasy FFPE Kit. The mRNA was reverse-transcribed to cDNA at 42 °C for 1 h. β-actin was used as the internal control. The RT-PCR conditions were as follows: an initial denaturation at 95 °C for 5 min, followed by 95 °C for 25 s, 64 °C for 20 s, and 72 °C for 20 s to ensure the specificity; and 31 cycles at 93 °C for 25 s, 60 °C for 35 s, 72 °C for 20 s were performed for data collection and sensitivity analysis. All of the positive genes including mutations or fusions were confirmed with Sanger sequencing. All the experiments were performed according to the user manual as described previously [22].

### Statistical analysis

The Chi squared test was used to evaluate the relationships between different driver genes and PD-L1 expression. Survival curves of pathologically confirmed samples were calculated using the Kaplan–Meier method until death or last follow-up. Multivariate analysis was performed using the Cox regression model. Statistical analysis was performed with the SPSS 18 software (Chicago, IL, USA). *P* < 0.05 was considered statistically significant. The median follow-up was 54 months (6.5–65) and the last follow-up date was July 31, 2015.

## Results

### Patient characteristics

Patients’ clinical profile is presented in Table 1. One hundred and ninety-eight patients (51.4 %) were male with a median age of 59 years. One hundred and fifty (39.0 %) patients were former or current smokers. Pathologic stage I was seen in 121 patients, stage II in 79 patients, and stage III in 185 Patients.

**Table 1:** Demographic characteristics of the study population (n = 385)

	Number
Gender	
Male	198
Female	187
Age	
Range	28–79
Median	59
<60	207
≥60	178
Smoking status	
Never	235
Former/current	150
Stage	
I	121
II	79
III	185
PD-L1 expression	
Yes	186
No	199
Gene alteration	
EGFR	205
ALK	18
KRAS	16
HER2	9
ROS1	8
PIK3CA	6
RET	6
BRAF	2
NRAS	1
Concurrent alteration	24
Pan-negative	90
Adjuvant treatment	
Yes	269
No	116

### Gene analysis results

All the patients were analyzed for *EGFR, KRAS, NRAS, PIK3CA, BRAF* and *HER2* mutations and *ALK, ROS1, RET* fusion genes. This analysis included EGFR mutations (n = 205, 53.2 %), followed by *EML4*–*ALK* rearrangements (n = 18, 4.7 %), *KRAS* (n = 16, 4.2 %), *HER2* (n = 9, 2.3 %), *ROS1* (n = 8, 2.1 %), *PIK3CA* (n = 6, 1.6 %), *RET* (n = 6, 1.6 %), *BRAF* (n = 2, 0.5 %), and *NRAS* (n = 1, 0.2 %), and 24 coexisting mutations (6.2 %). All the nine genes were negative in 90 patients, defined as pan-negative. The details of coexisting mutations are listed in Table 2.

**Table 2:** Clinical characteristics and PD-L1 expression in concurrent gene alteration patients

Case	Gender	Age	Stage	Smoking	Gene type	PD-L1 expression	OS (month)
1	Male	43	IB	Yes	EGFR+PIK3CA	Yes	67+
2	Female	51	IIIA	No	RET+PIK3CA	Yes	42
3	Female	58	IIIA	No	EGFR+ALK	Yes	34
4	Male	74	IA	No	EGFR+PIK3CA	No	66+
5	Male	60	IIIA	Yes	KRAS+ALK	Yes	35+
6	Female	60	IB	No	EGFR+RET-M2	Yes	54
7	Female	60	IA	No	EGFR+PIK3CA	Yes	36+
8	Male	64	IIA	No	RET+PIK3CA	No	55
9	Male	69	IB	No	KRAS6+HER2	Yes	43+
10	Male	45	IIIA	Yes	KRAS+PIK3CA	Yes	25
11	Female	64	IIB	Yes	EGFR+HER2	No	46+
12	Female	75	IIIA	No	EGFR+PIK3CA	No	24+
13	Female	69	IIA	No	KRAS+PIK3CA	Yes	36
14	Female	49	IB	No	EGFR+HER2	Yes	48+
15	Female	55	IIB	Yes	ROS1+HER2	Yes	37+
16	Male	62	IB	Yes	EGFR+ALK	No	46
17	Male	55	IIIA	No	EGFR+PIK3CA	Yes	39
18	Female	68	IB	No	EGFR+PIK3CA	Yes	58+
19	Female	76	IB	No	ALK+RET-M16	No	28
20	Male	43	IB	No	EGFR+PIK3CA	Yes	55+
21	Male	59	IIIA	Yes	KRAS+PIK3CA	Yes	18+
22	Female	61	IB	No	EGFR+PIK3CA	Yes	66+
23	Female	68	IIA	No	EGFR+HER2	Yes	45
24	Male	62	IIIA	Yes	KRAS+HER2	Yes	16

### PD-L1 expression correlated with driver genes

**Fig. 1:**
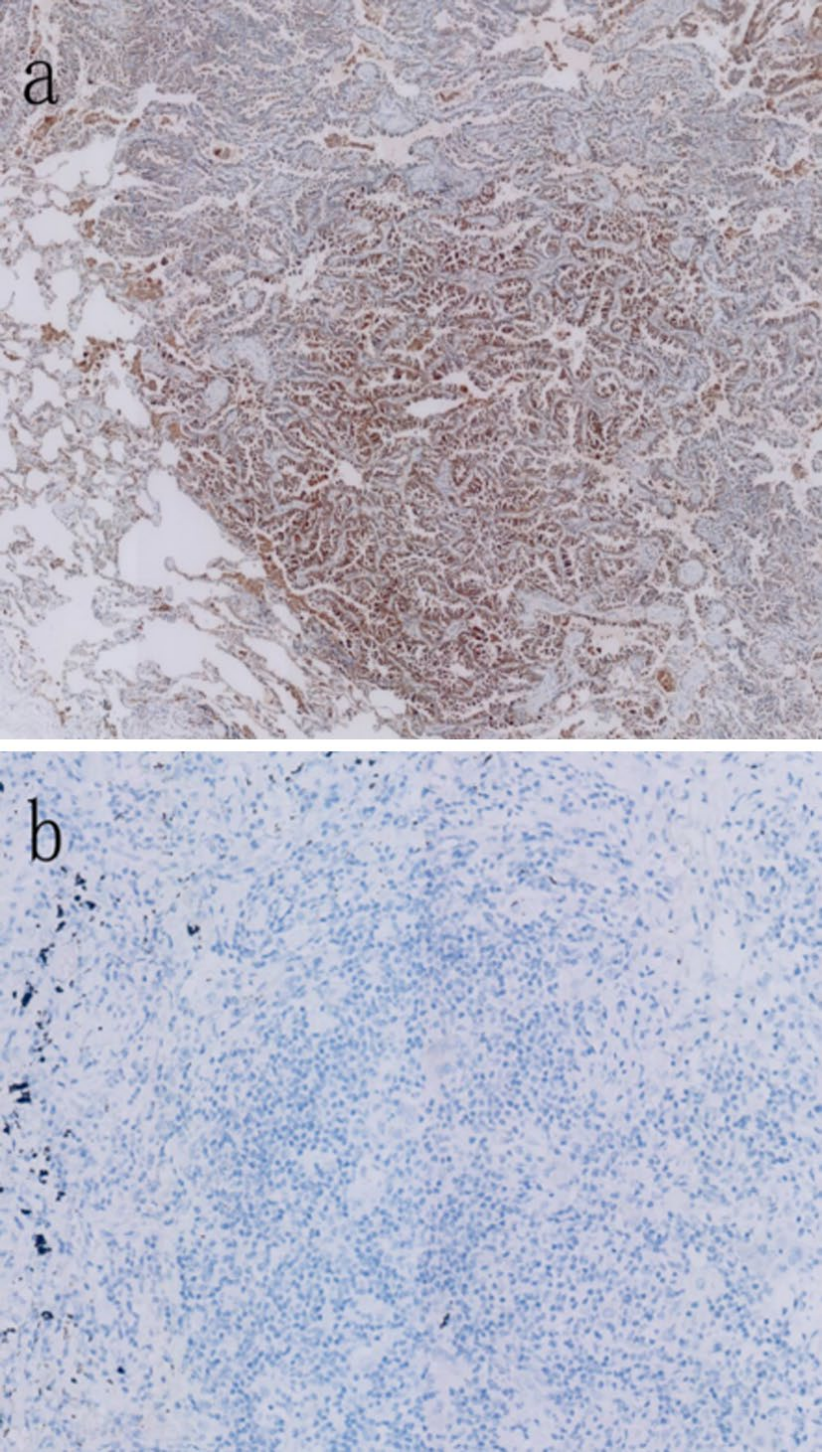
**a** Positive programmed cell death-ligand 1 (PD-L1) immunohistochemical staining in a patient with adenocarcinoma. **b** Negative PD-L1 immunohistochemical staining in another patient with adenocarcinoma

**Fig. 2:**
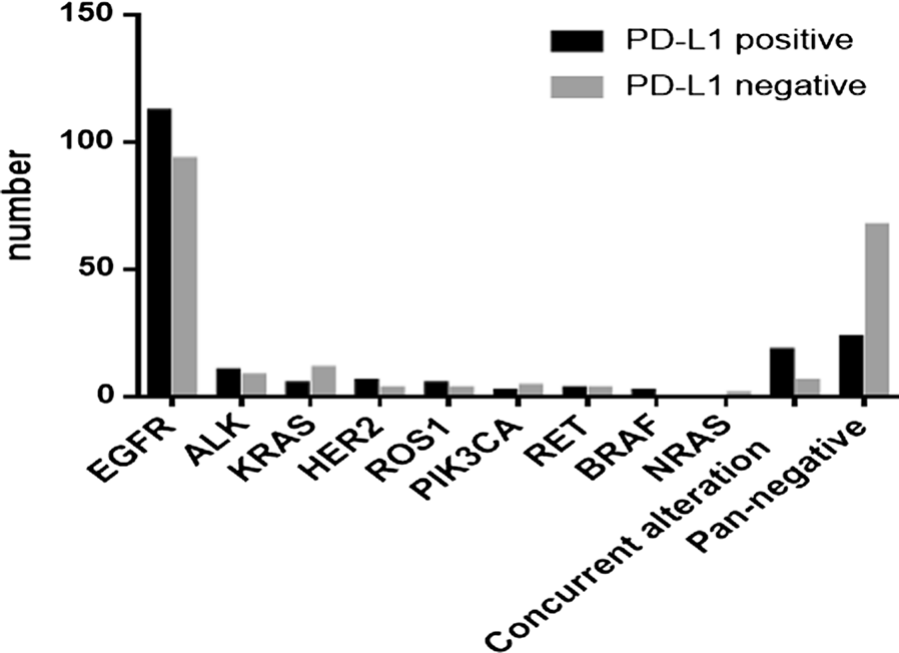
Relationship between PD-L1 expression and driver genes

The PD-L1 membrane expression was detected in 186 of the 385 lung adenocarcinoma patients (48.3 %) (Figs. 1, 2). The relationships between clinical parameters or gene characteristics and PD-L1 expression are shown in Table 3. PD-L1 expression was not significantly associated with any clinicopathologic parameters. Patients with PD-L1 positive expression more frequently presented with coexisting mutations (18/24, 75 %), followed by single-gene mutation (145/271, 53.5 %) and pan-negative (23/90, 25.6 %) genes. Differences in PD-L1 expression were found among the coexisting mutations, single-gene mutations and pan-negative genes (*P* < 0.001).

**Table 3:** Clinical characteristics comparison between PD-L1 positive and negative expression in NSCLC patients

Variables	PD-L1 positive (n = 186)	PD-L1 negative (n = 199)	*P*
Gender			0.07
Male	87	111	
Female	99	88	
Age			0.68
<60	102	105	
≥60	84	94	
Smoking status			0.12
Never	121	114	
Former/current	65	85	
Pathologic stage			0.09
I + II	105	95	
III	81	104	
EGFR			0.008
Yes	112	93	
No	74	106	
ALK			0.53
Yes	10	8	
No	176	191	
KRAS			0.16
Yes	5	11	
No	181	188	
HER2			0.44
Yes	6	3	
No	180	196	
ROS1			0.65
Yes	5	3	
No	181	196	
PIK3CA			0.74
Yes	2	4	
No	184	195	
RET			0.74
Yes	3	3	
No	183	196	
BRAF			0.45
Yes	2	0	
No	184	199	
NRAS			0.97
Yes	0	1	
No	186	198	
Concurrent alteration			0.01
Yes	18	6	
No	168	193	
Pan-negative			<0.01
Yes	23	67	
No	163	132	

Another antibody (5H1, Cell Signaling Technology, Beverly, MA, USA) was used in 102 patients to detect the PD-L1 expression. The same trend of PD-L1 expression difference existed in patients with different gene abnormality. The PD-L1 positive patitnets was more frequently carried coexisting mutations (5/8, 62.5 %), followed by single-gene positive (32/66, 48.5 %) and pan-negative mutations (10/28, 25.6 %)(*P* = 0.337).

### Survival analysis

**Fig. 3:**
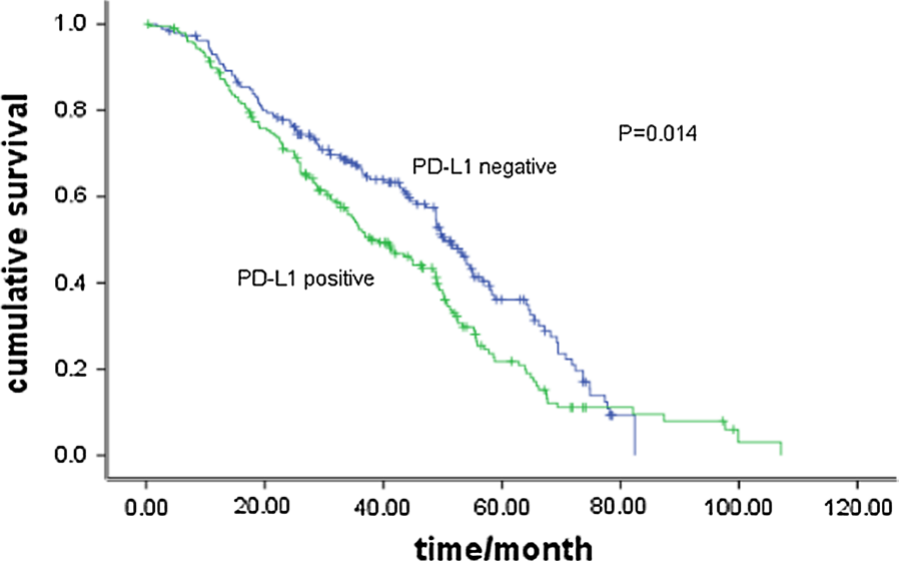
Disease free survival curves in patients with positive or negative programmed cell death-ligand 1 (PD-L1) staining (38.0 vs. 50.4 months, *P* = 0.014)

**Fig. 4:**
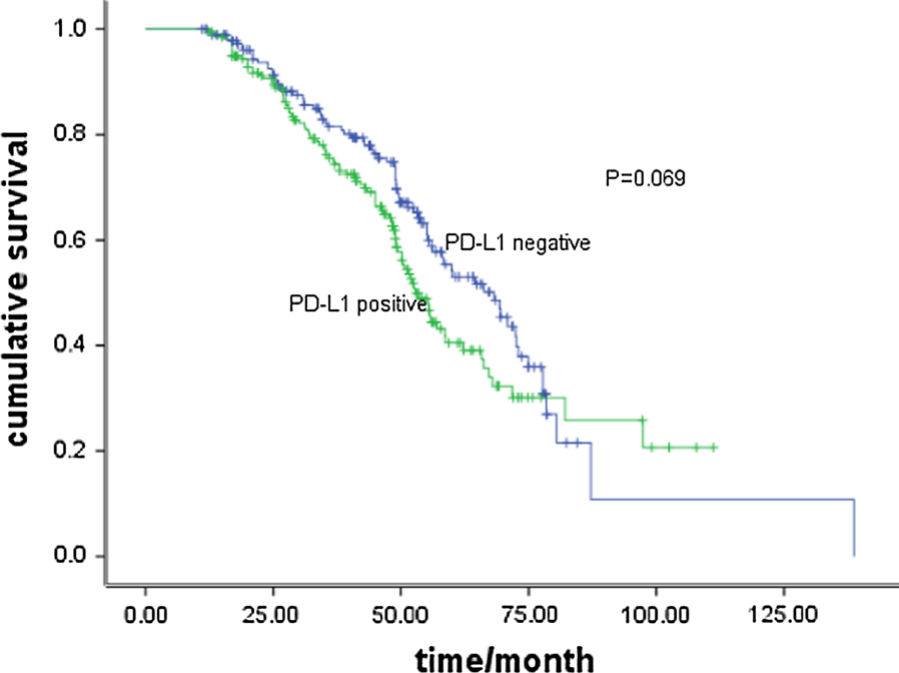
Overall survival curves in patients with positive or negative programmed cell death-ligand 1 (PD-L1) staining (52.9 vs. 68.2 months, *P* = 0.069)

The median DFS and OS were 48.3 and 58.1 months, respectively. Patients with positive PD-L1 expression had shorter DFS than those with negative PD-L1 expression (38.0 vs. 50.4 months, *P* = 0.014) (Fig. 3), but the OS between the two groups showed no significant difference (52.9 vs. 68.2 months, *P* = 0.069) (Fig. 4; Table 4).

**Table 4:** Univariate analysis for disease-free survival and overall survival

Variables	Median DFS	*P*	Median OS	*P*
Gender		0.74		0.44
Male	44.6		55.6	
Female	48.9		59.9	
Age		0.23		0.39
<60	49.3		59.3	
≥60	42.9		55.2	
Smoking status		0.16		0.59
Never	49.0		58.6	
Former/current	41.3		56.0	
Pathologic stage		<0.001		<0.001
I + II	52.5		66.2	
III	30.2		45.0	
Adjuvant treatment		0.54		0.76
Yes	49.7		59.2	
No	46.5		56.5	
Driver genes		0.23		0.24
Positive	48.9		58.7	
Negative	42.0		50.4	
PD-L1 expression		0.014		0.069
Yes	38.0		52.9	
No	50.4		62.0	

In univariate analysis, early stage (stage I and II versus III) and PD-L1 expression negative were significantly risk factors for tumor recurrence or metastasis (Figs. 3, 4), while only early stage was a favorable prognostic factor of OS (Table 4).

In multivariate analysis, only early stage suggested lower risk for DFS, while PD-L1 expression was not correlated with recurrence or metastasis. Early stage was an independent and favorable prognostic factor for OS (Table 5).

**Table 5:** Multivariate survival analysis for disease-free survival and overall survival

Variables	DFS			OS		
	HR	95 % CI	*P*	HR	95 % CI	*P*
Smoking status (smokers vs. non-smokers)	0.84	0.66–1.07	0.16	1.12	0.81–1.53	0.48
Stage (III vs. I + II)	1.71	1.32–2.21	0.00	1.16	0.84–1.58	0.00
PD-L1 expression (positive vs. negative)	1.17	0.91–1.51	0.22	1.79	1.30–2.46	0.37

## Discussion

This study shows that PD-L1 is overexpressed in 48.3 % (186/385) of lung adenocarcinoma patients and this overexpression is more frequently seen in patients with coexisting mutations, but less frequently in patients with pan-negative genes. The PD-L1 overexpression is not a prognostic factor for overall survival. To the best of our knowledge, this is the first study with the largest number of patients correlating the nine common driver genes in lung adenocarcinoma and PD-L1 expression.

Several studies have reported the association between *PD*-*L1* expression and driver genes [23, 24]. The results of the correlation were controversial. Azuma et al. [14] observed that *PD*-*L1* positive status was significantly associated with *EGFR* mutations . Mu et al. observed no significant correlation between *PD*-*L1* expression and *EGFR*/*ALK* status in stage I NSCLC patients [25]. Similarly, Zhang et al. found that no association between *PD*-*L1* expression and *EGFR* status in lung adenocarcinoma [25]. Therefore, the role of inhibition of PD-1/PD-L1 pathway and driver genes based on the results of existing studies is inconclusive, due to several reasons. First, most of the samples in previous studies were relatively small. Second, most of the studies focused on *EGFR* mutations or *ALK* rearrangements, and other driver genes were not well investigated. Last but not least, racial differences may play an important role in the controversial results.

In the present study, PD-L1 overexpression was more frequent in patients with coexisting mutations than in pan-negative patients. One explanation is that the genetic differences affected epigenetics, which may alter the expression of tumor-associated self-antigens, which in turn, affected tumor antigenicity. Increased number of driver genes reflects a higher level of neoantigens, which alters the immune microenvironment and increases the PD-L1 expression [26].

Because of heterogeneity of tumors, the efficacy of chemotherapy or molecular targeted treatment is relatively limited, combination treatment with different anti-cancer mechanisms drugs hold much potential in this area. Previous studies demonstrated that EGFR and ALK genes could induce PD-L1 expression to facilitate evasion of the host anti-tumour immune response, suggesting an active role for these genes in remodelling the immune microenvironment [27, 28]. In this way, combination of PD-1/PD-L1 blockade with targeted inhibitor or other drugs may be a promising therapeutic strategy to increase the duration of treatment response and delay development of drug resistance.

The role of PD-L1 in predicting the prognosis of NSCLC was controversial in previous studies [20]. Some studies found that negative PD-L1 expression led to superior OS in NSCLC patients compared with positive PD-L1 expression [14, 29], while Yang et al. [30] concluded that PD-L1 expression had no significant correlation with OS. In the present cohort, we found no association between the PD-L1 expression and overall survival in NSCLC patients. However, PD-L1 expression was related to shorter DFS. The results may contribute to the treatment after recurrence or metastasis.

Our study limitations are as follows. One major limitation was its retrospective nature. Second, only 24 patients with coexisting mutations were included, and the small sample size may influence the results of the current study. Third, different antibodies were used in different anti-PD-1 or PD-L1 therapies in clinical trials currently. The choice of antibody and the threshold for positivity might influence the results of different studies. Only one antibody and 5 % threshold were used in the present study. Different anti-PD-L1 antibodies may need to be validated in the same sample in future studies.

## Conclusions

In conclusion, we demonstrated the expression of PD-L1 in over 48 % of lung adenocarcinoma patients and the expression was associated with coexisting driver genes. PD-L1 expression is not associated with overall survival in patients with completely resected NSCLC.

## Abbreviations

PD-1: programmed death-1
NSCLC: non-small cell lung cancer
DFS: disease free survival
OS: overall survival
